# Enhancing carbapenem antimicrobial dosing optimization: synergy of antimicrobial stewardship teams and ward-based clinical pharmacists

**DOI:** 10.1017/ash.2024.30

**Published:** 2024-03-19

**Authors:** Tatsuya Tai, Takahiro Motoki, Kazunori Yamaguchi, Masahiro Watanabe, Taichi Ito, Kyoko Yokota, Kaori Ishikawa, Hiroaki Tanaka, Yuichi Muraki, Shinji Kosaka, Teruki Dainichi

**Affiliations:** 1 Department of Infection Control Service Office, Kagawa University Hospital, Kagawa, Japan; 2 Department of Pharmacy, Kagawa University Hospital, Kagawa, Japan; 3 Department of Pharmacology, School of Pharmacy, Shujitsu University, Okayama, Japan; 4 Infectious Disease Education Center, Kagawa University Hospital, Kagawa, Japan; 5 Department of Cardiorenal and Cerebrovascular Medicine, Kagawa University Hospital, Kagawa, Japan; 6 Laboratory of Clinical Pharmacoepidemiology, Kyoto Pharmaceutical University, Kyoto, Japan; 7 Department of Dermatology, Kagawa University Faculty of Medicine, Kagawa, Japan

## Abstract

Antimicrobial-product package inserts and insufficient staffing impede routine carbapenem monitoring in the inpatient setting in Japan. The collaboration between antimicrobial stewardship teams and clinical pharmacists was associated with a sustained improvement in carbapenem dosing optimization. Our findings could be of use to countries with inadequate monitoring of carbapenem antimicrobial use.

## Introduction

Multidrug-resistant and carbapenem-resistant bacteria pose a global threat, with Japan prioritizing antimicrobial stewardship (AS) for carbapenems.^1^ Proper administration of carbapenems is crucial for improved resistance rates and patient outcomes.^2^ In contrast, in Japan, antimicrobial package inserts may specify lower than Western standards and could lack information on renal-adjusted dosages. Hence, physicians may prescribe reduced doses for severe infections or adhere to standard antimicrobial prescriptions for patients with impaired renal function. In addition, AS activities in Japan being primarily implemented through programs centered on prospective audit and feedback, there is a notable shortage of AS staff for frequent monitoring of antibiotic therapy, adding to the challenge.^3^

Pharmacists usually optimize antimicrobial schedules.^2,4^ AS activities optimize carbapenems, reducing hospitalization duration by using ward-based clinical pharmacists’ (WBCPs) checklists.^5^ Collaboration between the antimicrobial stewardship team (AST) and WBCPs improves de-escalation rates.^4^ Only one study has been conducted on the impact of the collaborative system; it focused solely on the period 8-month post-introduction,^4^ without implementing interrupted time series analysis (ITSA).

We evaluated the impact of Kagawa University Hospital’s collaborative system (introduced on July 1, 2018), in which the AST and WBCPs participate in optimizing carbapenem dosage and administration schedules.

## Methods

### Setting

Kagawa University Hospital is a tertiary emergency care facility with 613 beds and 33 medical departments. The hospital’s AST, formed in 2013, comprises 1 nurse, 1 physician, 2 pharmacists, 1 clinical laboratory technician, and 1 administrative staff member. The carbapenems used here were meropenem, doripenem, and imipenem/cilastatin.

### Study design

This single-center study, conducted from January 1, 2016, to December 31, 2022, compared the pre-collaborative (January 2016 to June 2018) with the post-introduction (July 2018 to December 2022) phases, guided by the AST and WBCPs, targeting all cases at our institution who received carbapenem.

### Evaluation of the carbapenem dosage and administration schedule

Recommended schedules are described in Tables S1–S3. In this case, all administrations were intermittent. Non-recommended schedules deviate from the recommended dosages and schedules. Excessive intake is defined as deviations like “dosing interval shorter” or “single dose exceeding.” The primary outcome was compliance with the recommended schedule. Variables analyzed included the correction rate within 3 days, assessed using ITSA with a retrospective quasi-experimental design.

### AST activities in the pre-collaborative system

In the pre-collaborative system, the only AS requested information about the reason for carbapenem use and the causative microorganism upon initial prescription and after 10 days of administration. AST pharmacists thus maintained a weekly case list and informed physicians about any deviations from recommended dosages and schedules (Figure S1, Tables S1–S3).

### AST activities after introducing the collaborative system

Following the introduction of the collaborative system, AST pharmacists increased the case list daily and developed a template in the electronic medical-record system to monitor carbapenem dosage and administration schedules (Figure S1). The WBCPs, rather than the AST, offered feedback to attending physicians within 3 days if schedules deviated from the recommended carbapenem dosage and administration schedule guidelines.

### Evaluation of adverse events in cases of carbapenem overdose

Adverse events (AEs) in cases of carbapenem overdose were assessed according to the criteria of the Japanese Society of Chemotherapy’s Antimicrobial Safety Evaluation Committee, based on the Common Terminology Criteria for Adverse Events.^6^ Evaluation involved comparing events that occurred during drug administration before and after introduction of the collaborative system. Laboratory values identified as AEs are presented in Table S4.

### Statistical analysis

We used Fisher’s exact test to compare dosage and administration schedules before and after the introduction of the collaborative system. For ITSA, 2 periods were assessed: pre-introduction (January 2016 to June 2018) and post-introduction (July 2018 to December 2022). The model incorporated an intercept (β0), baseline trend (β1), change in level after system introduction (β2), and change in trend after system introduction (β3).^6^ Dependent variables were monthly rates of correcting non-recommended dosages within 3 days and compliance rate with recommended dosages. The post-introduction period was added as an independent variable. Significance was considered when *P* < .05. Statistical analysis utilized EZR version 1.32.

### Ethics statement

Informed consent was obtained from patients through online opt-out, adhering to Japan’s ethical guidelines for medical and biological research involving human subjects. The study followed the Clinical Guidelines for Medical Research Involving Humans and was approved by the Kagawa University Hospital Institutional Review Board (Approval No. 2022-085).

## Results

Most of the carbapenem prescriptions were for meropenem (94.5%; 2187/2315); doripenem was prescribed in 53 cases and imipenem/cilastatin in 75 cases (Figure S2). The collaborative system reduced the proportion of non-recommended schedules from 46.2% to 20.4% (*P* < .001, Table 1). The proportion of cases with a dose less than recommended decreased from 31.1% to 8.8% (*P* < .001), and those of cases with longer-than-recommended dosing intervals reduced from 5.1% to 2.9% (*P* = .011). After introduction of the collaborative system, the most common feedback involved meropenem 0.5 g every 8 hours (31.1%), followed by meropenem 0.5 g every 12 hours (20.6%) (Table 2).

**Table 1.** Comparison of dosage and administration schedule before and after the introduction of the collaborative system

Compliance status	Pre-introduction (*n*=820)	Post-introduction (*n*=1495)	*P* value
Recommended dosage and administration schedule	441 (53.8%)	1189 (79.5%)	<.001^a^
Non-recommended dosage and administration schedule			
Single dose less than the recommended	255 (31.1%)	132 (8.8%)	<.001^a^
Single dose more than the recommended	34 (4.1%)	58 (3.9%)	.740
Dosing interval shorter than recommended	48 (5.9%)	71 (4.7%)	.279
Dosing interval longer than recommended	42 (5.1%)	44 (2.9%)	.011^a^
Total	379 (46.2%)	305 (20.4%)	<.001^a^

a *P* < 0.05.

**Table 2.** Details of patients who received feedback after the introduction of the collaborative system

	Feedback *n*=210)
Age (in years)	75.0 (68.0–83.0)
Gender (number of patients; % male)	128 (61.2%)
Creatinine clearance (mL/min/1.73 m^2^)	45.0 (34.4–72.3)
Condition treated with carbapenems	
Sepsis	37 (17.7%)
Pneumonia	27 (12.9%)
Urinary tract infection	19 (9.1%)
Intra-abdominal infection	90 (43.1%)
Soft-tissue infection	15 (7.2%)
Meningitis	5 (2.4%)
Febrile neutropenia	7 (3.3%)
Other	8 (3.8%)
Dosage and treatment schedule before pharmacist’s intervention	
Meropenem 0.25 g every 12 h	1 (0.5%)
Meropenem 0.25 g every 24 h	5 (2.4%)
Meropenem 0.5 g every 8 h	65 (31.1%)
Meropenem 0.5 g every 12 h	43 (20.6%)
Meropenem 0.5 g every 24 h	4 (1.9%)
Meropenem 0.5 g every 48 h	1 (0.5%)
Meropenem 1 g every 8 h	42 (20.1%)
Meropenem 1 g every 12 h	30 (14.4%)
Meropenem 1 g every 24 h	4 (1.9%)
Meropenem 2 g every 8 h	5 (2.4%)
Meropenem 2 g every 12 h	6 (2.9%)
Meropenem 2 g every 24 h	1 (0.5%)
Imipenem/cilastatin 0.5 g every 8 h	2 (1.0%)
Imipenem/cilastatin 1 g every 12 h	1 (0.5%)

**Figure 1.**
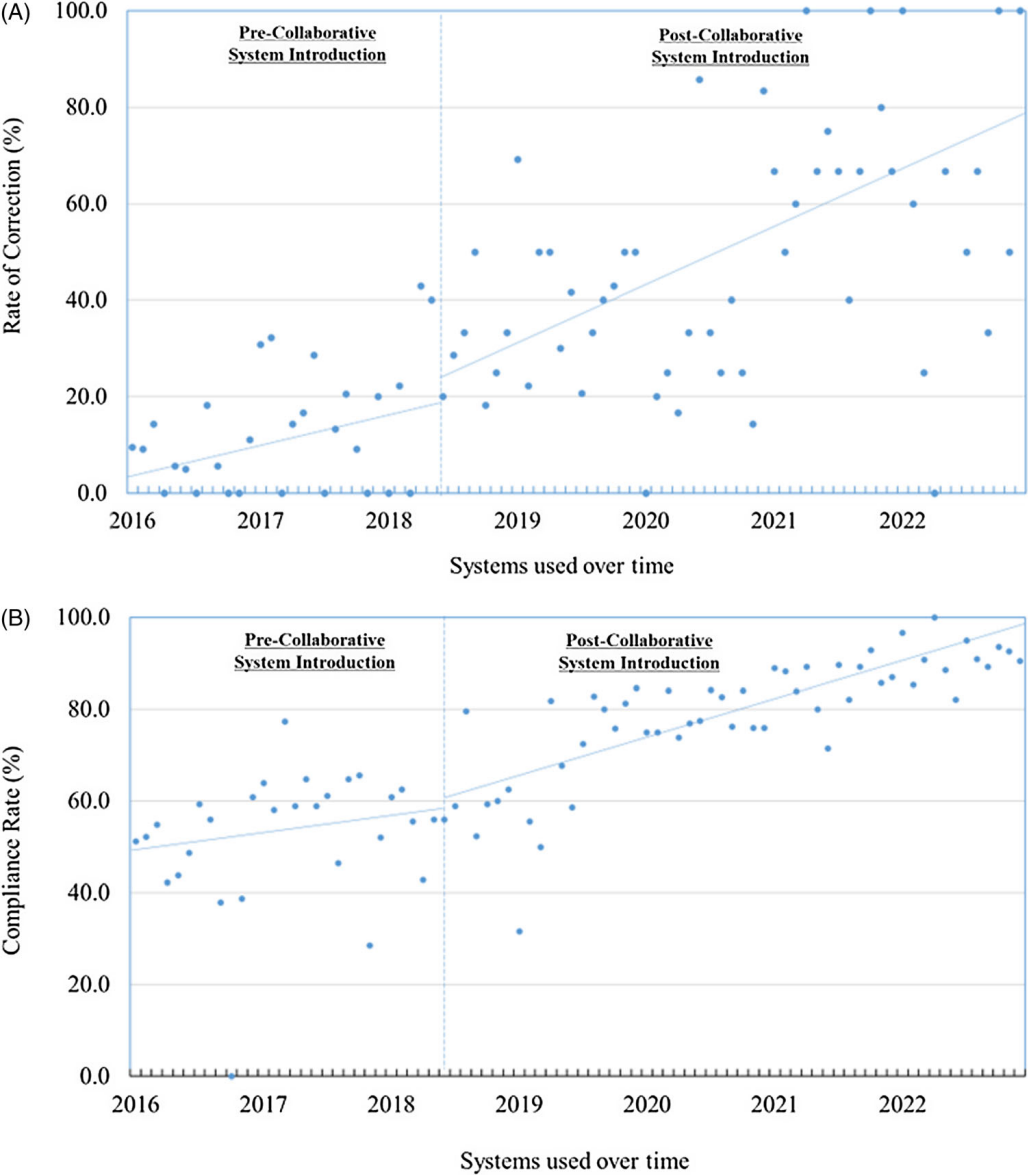
(A) Rate of correction within 3 days for non-recommended dosages and adherence to the recommended schedule over time and (B) trends over time in compliance with the recommended dosage and administration schedules of carbapenem antimicrobials, at Kagawa University Hospital.

The collaborative system led to a monthly increase of 0.96% (95% CI: 0.62–1.31; *P* < .001) in correcting non-recommended schedules within 3 days. However, immediate changes were not observed, with a level change of 3.98% (95% CI: –13.63–21.59; *P* = .65) (Figure 1A). The collaborative system resulted in a monthly increase of 0.66% (95% CI: 0.48–0.85; *P* < .001) in adherence to recommended carbapenem guidelines. Again, no immediate changes were observed, with a level change of 3.32% (95% CI: –6.39–13.02; *P* = .50) (Figure 1B).

The rates of carbapenem antibiotic overdose before and after introduction of the collaborative system did not differ significantly, with no severe symptoms like acute hepatitis or drug-related encephalopathy were reported (Table S5).

## Discussion

The collaborative system was associated with the lower proportion of non-recommended carbapenem dosage and schedules, especially for sub-recommendation dosing, without affecting the rate of carbapenem-related AEs. This results in being consistent with the past finding.^4^ As new knowledge, ITSA results demonstrated a sustained 54-month impact on the optimization of carbapenem dosage and administration.

The approved typical meropenem dosage for Japanese adults is 0.5 g every 8 hours, deemed non-recommended as it is half the Western standard. Consequently, non-recommended single dosing occurs frequently in Japan.^7^ Differences in package insert information between Japan and Western countries have broader implications,^8^ possibly affecting drug appropriateness.

Meropenem administered at dosages suitable for severe infections primarily led to liver dysfunction (9.5%).^7^ When meropenem was administered for purulent meningitis at double the dosage for severe infections, liver dysfunction (33.3%) was reported.^7^ This study identified mainly non-severe, liver-related AEs, consistent with earlier findings.^7^ Nevertheless, it is prudent to exercise vigilant monitoring of AEs going forward.

Effective AS feedback requires a trusting relationship between WBCPs and attending physicians, whether individualized or involving consultation.^4,5^ WBCPs, being already connected with physicians, can effectively address issues beyond infections, such as antipsychotic side effects and polypharmacy management in diabetes.^9^ Our study suggests that WBCPs could play an important role in the AST.

As this was a single-center study, it did not assess case outcomes or resistance emergence; future research should encompass data from multiple facilities for wider applicability. This study investigated the effectiveness of direct pharmacist intervention in enhancing carbapenem-antimicrobial dosing optimization.

In conclusion, the 54-month ITSA evaluation suggests that in regions with insufficient monitoring of AST and WBCP collaboration for carbapenem dosage compliance, a collaborative approach may address routine monitoring challenges, maintaining feedback quality, compared to traditional AS activities. It could potentially alleviate AS staff shortages.

## Supporting information

Tai et al. supplementary material 1 Tai et al. supplementary material

Tai et al. supplementary material 2 Tai et al. supplementary material

## Data Availability

Data are available upon request, subject to privacy, ethics, and other restrictions.

## Supplementary material

The supplementary material for this article can be found at https://doi.org/10.1017/ash.2024.30.
