# Supplementary material for: Enhancing carbapenem antimicrobial dosing optimization: synergy of antimicrobial stewardship teams and ward-based clinical pharmacists
Source: Antimicrob Steward Healthc Epidemiol. 2024 Mar 19;4(1):e33. doi: 10.1017/ash.2024.30 (PMC10964187; doi:10.1017/ash.2024.30)
Supplement: Tai et al. supplementary material 1 — Tai et al. supplementary material [file S2732494X24000305sup001.pptx]

## Slide 1
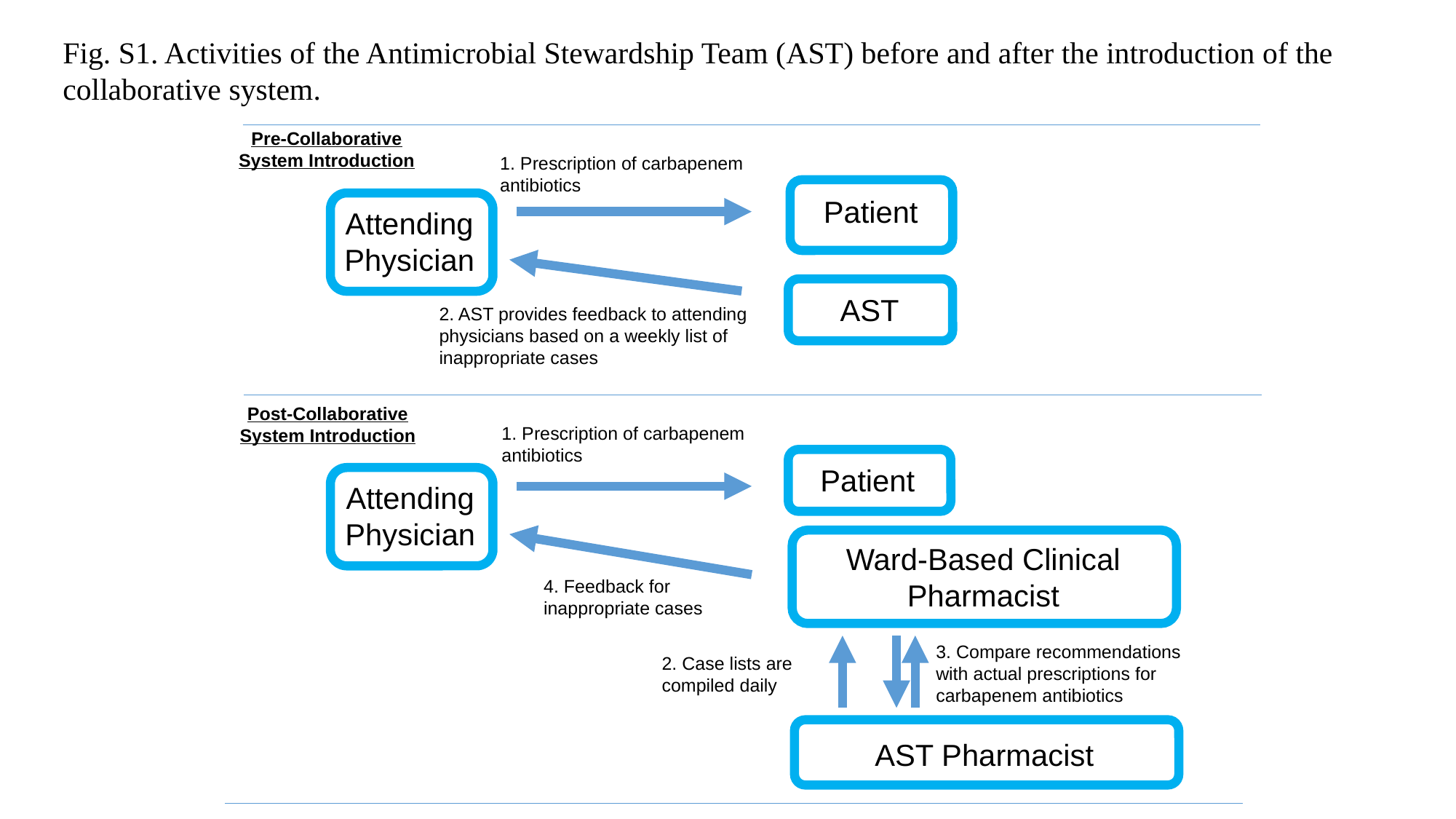

Fig. S1. Activities of the Antimicrobial Stewardship Team (AST) before and after the introduction of the collaborative system.
Pre-Collaborative System Introduction
1. Prescription of carbapenem antibiotics
Patient
Attending Physician
AST
2. AST provides feedback to attending physicians based on a weekly list of inappropriate cases
Post-Collaborative System Introduction
1. Prescription of carbapenem antibiotics
Patient
Attending Physician
Ward-Based Clinical Pharmacist
4. Feedback for inappropriate cases
3. Compare recommendations with actual prescriptions for carbapenem antibiotics
2. Case lists are compiled daily
AST Pharmacist

## Slide 2
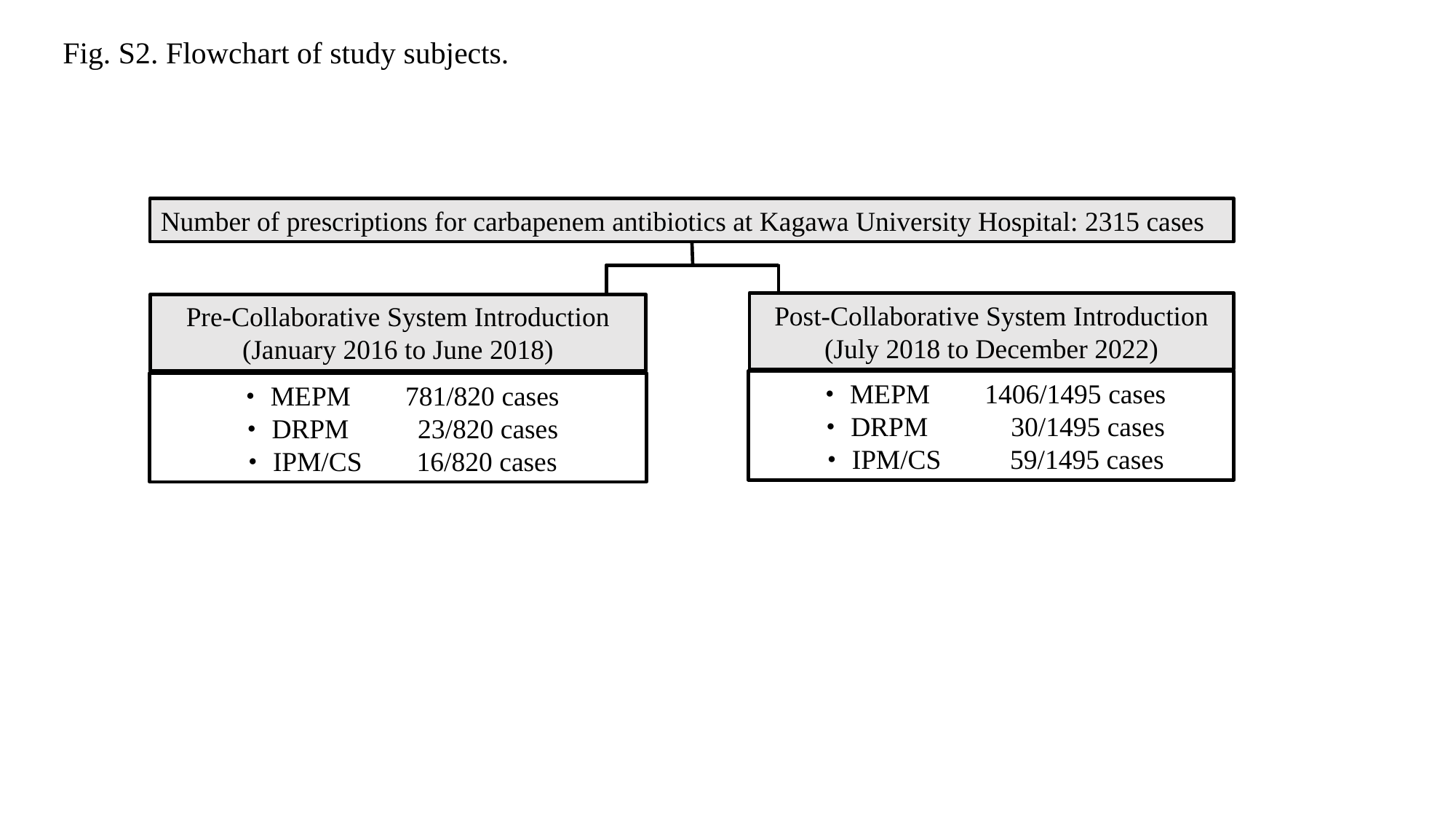

Fig. S2. Flowchart of study subjects.
Number of prescriptions for carbapenem antibiotics at Kagawa University Hospital: 2315 cases
Post-Collaborative System Introduction (July 2018 to December 2022)
Pre-Collaborative System Introduction (January 2016 to June 2018)
・MEPM　 1406/1495 cases
・DRPM　 30/1495 cases
・IPM/CS　 59/1495 cases
・MEPM　 781/820 cases
・DRPM　 23/820 cases
・IPM/CS　 16/820 cases

## Slide 3
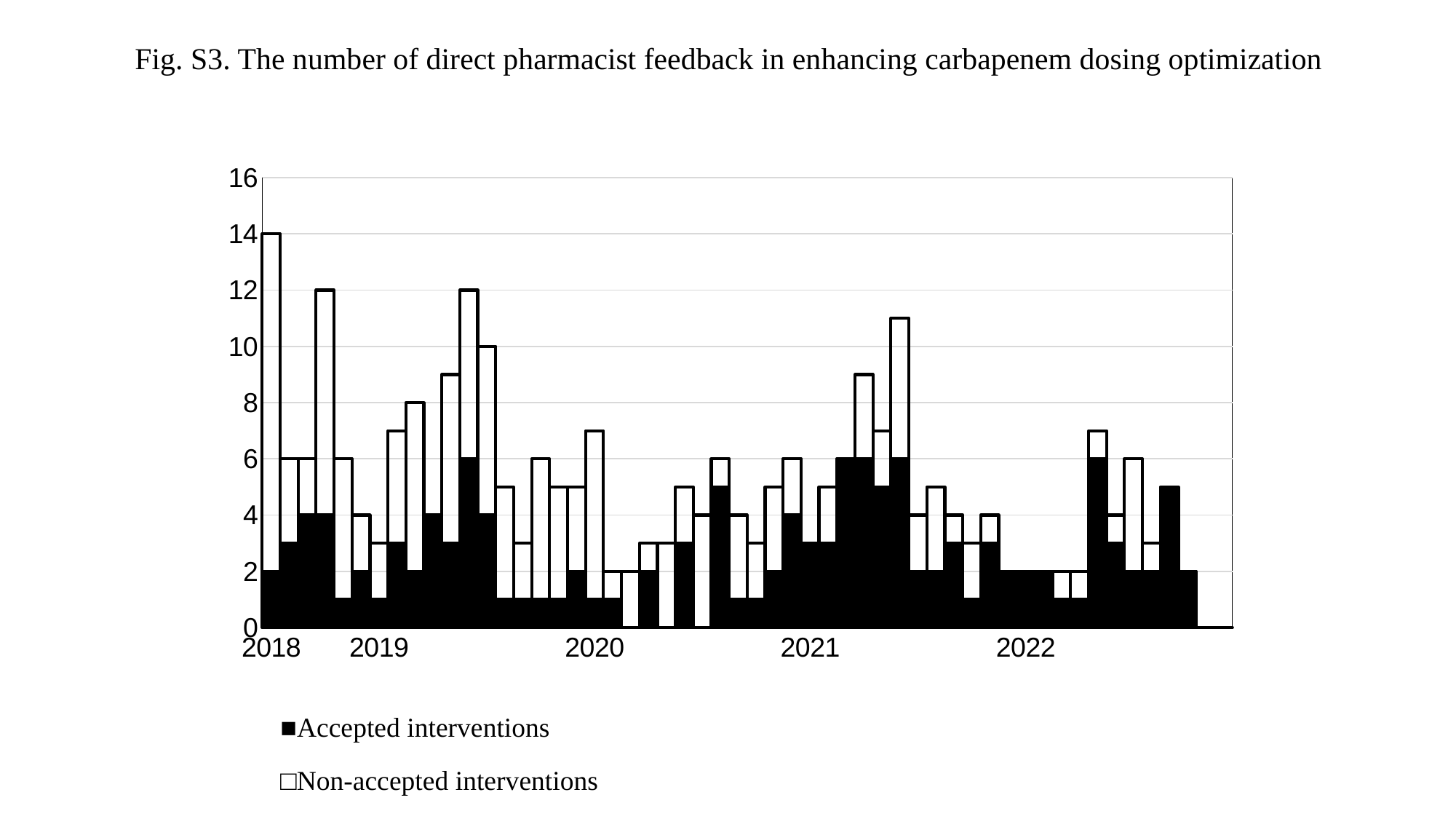

Fig. S3. The number of direct pharmacist feedback in enhancing carbapenem dosing optimization
### Chart
| Category | 受諾された介入 | 受諾されなかった介入 |
|---|---|---|
| 2018 | 2.0 | 12.0 |
| | 3.0 | 3.0 |
| | 4.0 | 2.0 |
| | 4.0 | 8.0 |
| | 1.0 | 5.0 |
| | 2.0 | 2.0 |
| 2019 | 1.0 | 2.0 |
| | 3.0 | 4.0 |
| | 2.0 | 6.0 |
| | 4.0 | 0.0 |
| | 3.0 | 6.0 |
| | 6.0 | 6.0 |
| | 4.0 | 6.0 |
| | 1.0 | 4.0 |
| | 1.0 | 2.0 |
| | 1.0 | 5.0 |
| | 1.0 | 4.0 |
| | 2.0 | 3.0 |
| 2020 | 1.0 | 6.0 |
| | 1.0 | 1.0 |
| | 0.0 | 2.0 |
| | 2.0 | 1.0 |
| | 0.0 | 3.0 |
| | 3.0 | 2.0 |
| | 0.0 | 4.0 |
| | 5.0 | 1.0 |
| | 1.0 | 3.0 |
| | 1.0 | 2.0 |
| | 2.0 | 3.0 |
| | 4.0 | 2.0 |
| 2021 | 3.0 | 0.0 |
| | 3.0 | 2.0 |
| | 6.0 | 0.0 |
| | 6.0 | 3.0 |
| | 5.0 | 2.0 |
| | 6.0 | 5.0 |
| | 2.0 | 2.0 |
| | 2.0 | 3.0 |
| | 3.0 | 1.0 |
| | 1.0 | 2.0 |
| | 3.0 | 1.0 |
| | 2.0 | 0.0 |
| 2022 | 2.0 | 0.0 |
| | 2.0 | 0.0 |
| | 1.0 | 1.0 |
| | 1.0 | 1.0 |
| | 6.0 | 1.0 |
| | 3.0 | 1.0 |
| | 2.0 | 4.0 |
| | 2.0 | 1.0 |
| | 5.0 | 0.0 |
| | 2.0 | 0.0 |
| | 0.0 | 0.0 |
| | 0.0 | 0.0 |■Accepted interventions
□Non-accepted interventions
