# Supplementary material for: Enhancing carbapenem antimicrobial dosing optimization: synergy of antimicrobial stewardship teams and ward-based clinical pharmacists
Source: Antimicrob Steward Healthc Epidemiol. 2024 Mar 19;4(1):e33. doi: 10.1017/ash.2024.30 (PMC10964187; doi:10.1017/ash.2024.30)
Supplement: Tai et al. supplementary material 2 — Tai et al. supplementary material [file S2732494X24000305sup002.docx]

Table S1. Recommended dosage and administration schedule of meropenem ^a^

| Creatinine clearance (mL/min) | Dose | Frequency |
| --- | --- | --- |
| >50 | 1 g | Every 8 hours |
| ca. 50-25 | 1 g | Every 12 hours |
| ca. 25-10 | 0.5 g | Every 12 hours |
| <10 | 0.5 g | Every 24 hours |
| Hemodialysis | 0.5 g | Every 24 hours (administered after dialysis on dialysis days) |
| Continuous ambulatory peritoneal dialysis | 0.5 g | Every 24 hours |
| Continuous renal replacement therapy | 1 g | Every 12 hours |

Non-recommended schedules are those that deviate from the recommended dosages and schedules.

For conditions other than pediatric meningitis: Administer 30–60 mg/kg per day in three divided doses (adjusting as needed based on age and symptoms). In severe or refractory infections, the dosage may be increased to 120 mg/kg per day. However, the daily maximum should not exceed 3 g in adults.

For pediatric meningitis: Administer 120 mg/kg per day in three divided doses (adjusting as needed based on age and symptoms). However, the daily maximum should not exceed 6 g in adults.

For pediatric febrile neutropenia: Administer 120 mg/kg per day in three divided doses (adjusting as needed based on age and symptoms). However, the daily maximum should not exceed 3g in adult

^a^In cases of meningitis, all doses are doubled.

Table S2. Recommended Dosage and Administration Schedule of Doripenem

| Ccr (mL/min) | Daily Dose Corresponding to the Daily Dose for Patients with Normal Renal Function (Ccr ≥ 70) | | | | | | | |
| --- | --- | --- | --- | --- | --- | --- | --- | --- |
|  | Dose | Frequency | Dose | Frequency | Dose | Frequency | Dose | Frequency |
| 70 > Ccr ≥ 50 | 0.25 g | Every 12 hours | 0.25 g | Every 8-12 hours | 0.5 g | Every 8-12 hours | 1.0 g | Every 12 hours ^a^ |
| 50 > Ccr ≥ 30 | 0.25 g | Every 12 hours | － | － | 0.25 g or 0.5 g | Every 8-12 hours | 0.5 g | Every 8 hours |
| 30 > Ccr ^c^ | － | － | 0.25 g | Every 12 hours ^b^ | － | － | 0.25 g | Every 8 hours ^b^ |

Ccr, creatinine clearance.

Non-recommended schedules are those that deviate from the recommended dosages and schedules.

For pediatric patients: Administer 60 mg/kg per day in three divided doses (adjusting as needed based on age and symptoms). In severe or refractory infections, the dosage may be increased up to 120 mg/kg per day. However, the daily maximum should not exceed 3 g in adults.

^a^ It is desirable to avoid administering a total of 1.0 g in every 8 hours.

^b^ Administer with caution in patients with low body weight, prioritizing safety.

^c^ For patients with Ccr < 10, consider switching to alternative medications in the absence of data.

Table S3. Recommended Dosage and Administration Schedule of imipenem/cilastatin

| Ccr (ml/min) | Adjustment Based on Dosage | | Adjustment Based on Dosing Interval | |
| --- | --- | --- | --- | --- |
|  | Dose | Frequency | Dose | Frequency |
| >50 ^a^ | 0.5g | Every 12 hours | 0.5g | Every 12 hours |
| 50 to 30 | 0.25g-0.5g | Every 12 hours | 0.5g | Every 12-24 hours |
| 30 to 10 ^b^ | 0.125g-0.25g | Every 12 hours | － | － |

Ccr, creatinine clearance.

Non-recommended schedules are those that deviate from the recommended dosages and schedules.

For pediatric patients: Administer 30–80 mg/kg per day in three to four divided doses (adjusting as needed based on age and symptoms). In severe or refractory infections, the dosage may be increased up to 100 mg/kg per day.

^a^ For severe or refractory infections, the daily dose can be increased to 2.0 g (1.0 g every 12 hours).

^b^ Because of the risk of convulsive seizures in patients with renal impairment, consider switching to other medications.

Table S4. Criteria for categorizing abnormal differences in laboratory values as adverse events

| Laboratory test | Criteria for categorization as adverse events |
| --- | --- |
| Liver dysfunction  ・Aspartate aminotransferase (AST)  ・Alanine aminotransferase (ALT)  ・γ-Glutamyl transferase (γ-GT)  ・Alkaline phosphatase (ALP)  ・Lactate dehydrogenase (LDH) | 1.5-fold of the upper limit of the institutional standard levels |
| Kidney dysfunction  ・Serum creatinine  ・Blood urea nitrogen (BUN) | Above 1.5-fold of the upper limit of the institutional standard levels |
| Leukopenia  ・White blood cell count (WBC) | <3000 cells/mm^3^ |
| Drug-related encephalopathy | Mild symptoms |

Table S5. Evaluation of adverse events in cases of carbapenem antibiotic overdose before and after the introduction of the collaborative system

|  | Pre-Introduction (n=82) | Post-Introduction  (n=129) | *P*-value |
| --- | --- | --- | --- |
| Liver dysfunction | 14 (17.1%) | 22 (17.1%) | 1.000 |
| Kidney dysfunction | 0 (0%) | 1 (0.8%) | 1.000 |
| Leukopenia | 5 (6.1%) | 3 (2.3%) | 0.266 |
| Drug-related encephalopathy | 0 (0%) | 0 (0%) | 1.000 |
| Cases exhibiting any adverse event^a^ | 17 (20.7%) | 25 (19.4%) | 0.860 |
| Cases not exhibiting any adverse event | 65 (79.3%) | 104 (80.6%) | 0.860 |

^a^ Some patients experienced more than one adverse event
